# Accelerated gene evolution and subfunctionalization in the pseudotetraploid frog *Xenopus laevis*

**DOI:** 10.1186/1741-7007-5-31

**Published:** 2007-07-25

**Authors:** Uffe Hellsten, Mustafa K Khokha, Timothy C Grammer, Richard M Harland, Paul Richardson, Daniel S Rokhsar

**Affiliations:** 1 Department of Energy Joint Genome Institute, 2800 Mitchell Drive, Walnut Creek, CA 94598, USA; 2 Center for Integrative Genomics and Department of Molecular and Cell Biology, University of California at Berkeley, Berkeley, CA 94720, USA

## Abstract

**Background:**

Ancient whole genome duplications have been implicated in the vertebrate and teleost radiations, and in the emergence of diverse angiosperm lineages, but the evolutionary response to such a perturbation is still poorly understood. The African clawed frog *Xenopus laevis *experienced a relatively recent tetraploidization ~40 million years ago. Analysis of the considerable amount of EST sequence available for this species together with the genome sequence of the related diploid *Xenopus tropicalis *provides a unique opportunity to study the genomic response to whole genome duplication.

**Results:**

We identified 2218 gene triplets in which a single gene in *X. tropicalis *corresponds to precisely two co-orthologous genes in *X. laevis *– the largest such collection published from any duplication event in animals. Analysis of these triplets reveals accelerated evolution or relaxation of constraint in the peptides of the *X. laevis *pairs compared with the orthologous sequences in *X. tropicalis *and other vertebrates. In contrast, single-copy *X. laevis *genes do not show this acceleration. Duplicated genes can differ substantially in expression levels and patterns. We find no significant difference in gene content in the duplicated set, versus the single-copy set based on molecular and biological function ontologies.

**Conclusion:**

These results support a scenario in which duplicate genes are retained through a process of subfunctionalization and/or relaxation of constraint on both copies of an ancestral gene.

## Background

Gene duplication followed by subsequent functional divergence is widely recognized as an important mechanism for the evolution of novelty [1,2]. On a small scale, local tandem duplications can rapidly produce new gene families, such as the Hox cluster in animals [3], the olfactory receptors in vertebrate genomes [4], and numerous other examples in plants [5,6], protists [7] and other lineages. Recently duplicated genes have a strong tendency to become pseudogenes, and will generally be lost due to disabling mutations unless positive selection preserves the duplicate loci. Based on the divergence of surviving gene pairs in diverse genomes, the typical lifetime of duplicated genes in a diploid background has been estimated to be several million years [8].

On a grander scale, entire genomes can be duplicated by polyploidization so that the cells of the resulting organism find themselves with two copies of every gene. Again, there is presumably a strong tendency towards rapid differential loss due to mutation of superfluous copies, and the long-term effect on the genome is elimination of most of the duplicate loci [9]. In the case of polyploidy, the population dynamic and stoichiometric effects are different from the case of a localized duplication in a diploid background. Loss of a copy of a locally-duplicated gene simply restores the pre-duplication genome. In contrast, in the case of whole genome duplication the polyploid population is presumably reproductively isolated from its diploid brethren, and inactivation/loss of one of a pair of duplicate sequences puts that gene at half the copy number of the remaining loci, at least in the early stages of rediploidization. As haploinsufficiency is relatively rare [10], reduced copy number is not by itself an overwhelming impediment to large scale loss, as is evident from analysis of surviving duplicates in the *Arabidopsis*, rice, teleost, and yeast genomes [9,11-13].

Early thoughts on the selective forces leading to duplicate gene retention centered on divergence in protein function. This suggests that one or both copies could acquire novel [1] and/or complementary [14] biochemical functions that would render both copies indispensable. It was further recognized that novel or complementary organismal functions could arise from differential regulatory mutations [14,15]. Thus, if duplicate genes become expressed in different cell types or developmental stages, they might become indispensable and resistant to loss even if their associated peptides remain interchangeable. Through this mechanism, novel spatiotemporal roles can emerge, with numerous individual examples of *cis*- or *trans-*regulatory subfunctionalization known, for example, in teleost fish [13].

The well-studied amphibian *Xenopus laevis *has chromosome number (2N = 36) and genome size (~3Gb), roughly double that of its congener *Xenopus *(formerly *Silurana*) *tropicalis *(2N = 20, ~1.5 Gb) [16,17]. This difference is attributed to a merger of two diploid progenitors originating ~40 million years ago [16,18-20]. Allotetraploidy is suggested by the ease with which modern *Xenopus *species can form hybrids via unreduced gametes [18]. However, we cannot rule out an autotetraploid origin. In this latter case, the duplicated pairs would be identical at the duplication event, whereas in the allotetraploid case such pairs would represent orthologs from the speciation event of the progenitors and might have separated at slightly different epochs prior to their last common ancestor, depending on the level of polymorphism at speciation. However, the differences in measurable terms are subtle, and in the following we refer to polyploidization events as genome duplications regardless of their origin. The *X. laevis *genome duplication is significantly more recent than the teleost-specific duplication (~350 million years ago (Mya)) [11,21] and the ancient vertebrate-specific duplications (> 500 Mya) [22,23]. However, it is older than the typical lifetime of duplicated genes in a diploid background (several million years) [8]. Thus, by comparing *X. laevis *and *X. tropicalis *gene pairs, we can analyze an animal gene complement relatively soon after rediploidization, taking advantage of large-scale genome sequence data.

## Results and Discussion

To study the evolution of duplicate gene pairs in *X. laevis *relative to their unique orthologs in *X. tropicalis*, we identified 20223 *X. laevis *open reading frames (ORFs) from an assembly of over half a million expressed sequence tags (ESTs) and transcripts [24], and compared them with each other and with a set of 24957 predicted transcripts from the *X. tropicalis *genome project (PM Richardson et al, unpublished results). Over half of the *X. laevis *ORFs in our set appear to be complete – that is, with a plausible start and stop codon.

**Figure 1:**
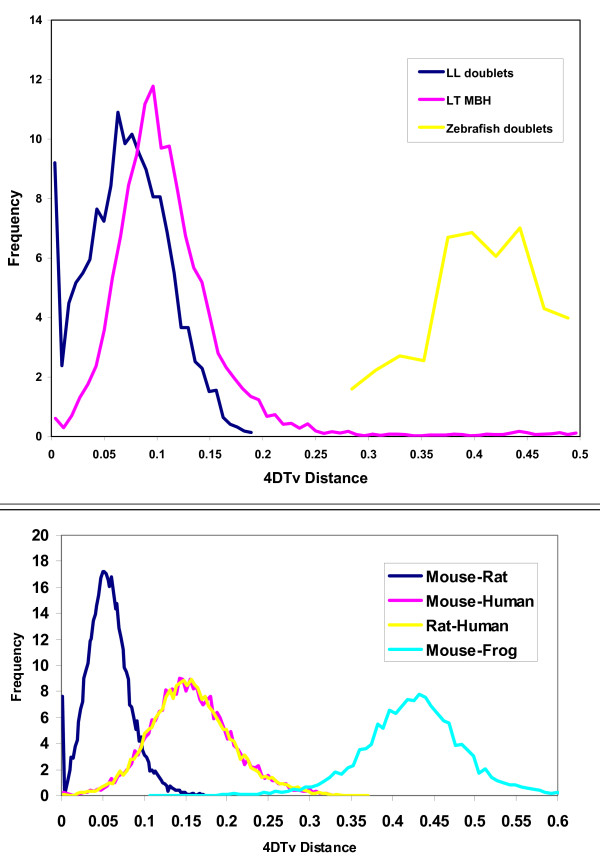
**Four-fold synonymous transversion rates**. (a) *X. tropicalis*-*X. laevis *mutual-best hits (LT MBH) show 4 DTv distances sharply peaked around 0.09 corresponding to the species divergence. The few hits in the high-end tail (4 DTv > 0.2) are due to the incompleteness of the gene sets and/or gene losses. The line marked LL doublets shows two-member clusters of recent (4 DTv < 0.15) *X. laevis *paralogs. Assuming uniform transversion rates across vertebrates, and dating the last common human-mouse ancestor at 75 Mya, the *laevis*-*tropicalis *and *laevis*-*laevis *divergence is ~50 and ~40 Mya, respectively. For comparison, paralogs from the much more ancient teleost duplication in zebrafish are also shown. After correcting for multiple transversions, the fish duplication is about eight times older than the *X. laevis *event, consistent with timings based on total synonymous substitution rates [13,14]. (b) 4 DTv distributions for orthologs in mouse-rat (red), mouse-human (blue), rat-human (green), and mouse-*X. tropicalis *(purple). Only orthologs supported by conserved synteny are considered. Using the same molecular clock as panel (a), the mammal-frog divergence is 350 Mya.

To measure the evolutionary divergence between *X. laevis *and *X. tropicalis *orthologs, and between gene pairs within *X. laevis *arising from the whole genome duplication event (paralogs), we determined the transversion rate at four-fold synonymous codon positions, denoted 4 DTv (see Additional file 1). We used transversions rather than total substitutions as (a) they occur at a slower rate than transitions, (b) they provide a simpler molecular clock as no assumptions or modeling of transition/transversion rates are needed for multiple-substitution correction, and (c) transversions are insensitive to local variations in GC content and unaffected by methylation effects. Figure 1a shows that 4 DTv distributions are sharply peaked for both *X. laevis*-*X. tropicalis *intergenomic mutual best aligned pairs (LT) and for *X. laevis*-*X. laevis *intragenomic pairs (LL), consistent with synchronous gene divergence due to speciation and gene duplication, respectively. No corresponding recent peak was found in the *X. tropicalis *self comparison (data not shown). For comparison, the distributions of 4 DTv distances between mouse-rat, mouse-human, and mouse-frog orthologs are shown in Figure 1b.

From this analysis we identified 9574 likely *X. laevis*-*X. tropicalis *(LT) orthologous genes. A simple molecular clock estimate puts the divergence of the *X. laevis *and *X. tropicalis *lineages at ~50 Mya, and the genome duplication event at ~40 Mya, consistent with mitochondrial data [19] and a previous analysis of a dozen duplicated genes [25].

Guided by Figure 1, we conservatively identified pairs of *X. laevis *paralogs for 2218 of the LT genes. These define high confidence LLT triplets such that (a) the *X. laevis *pair arose during the whole genome duplication event and is retained in the modern pseudotetraploid genome within the expressed gene dataset, and (b) the single *X. tropicalis *gene is the unique ortholog. *X. laevis *paralogs are arbitrarily designated L1 and L2; both are "co-orthologs" [26] of the corresponding *X. tropicalis *gene. This set represents the largest collection of such triplets from any whole genome duplication event in animals – three to four times larger than in teleost fish [26,27] and four to five times larger than in previous work on *Xenopus *[28,29]. Zebrafish duplicates from the much older teleost genome duplication show near-saturation at the synonymous codon positions (Figure 1a) [27,30].

How many of the ancient duplicated *X. laevis *gene pairs have subsequently lost one of the copies? This number cannot be accurately determined with only a partial collection of *X. laevis *genes based on ESTs. Nevertheless, we can crudely estimate a likely loss range of 50–75%, as discussed in the Methods section.

**Figure 2:**
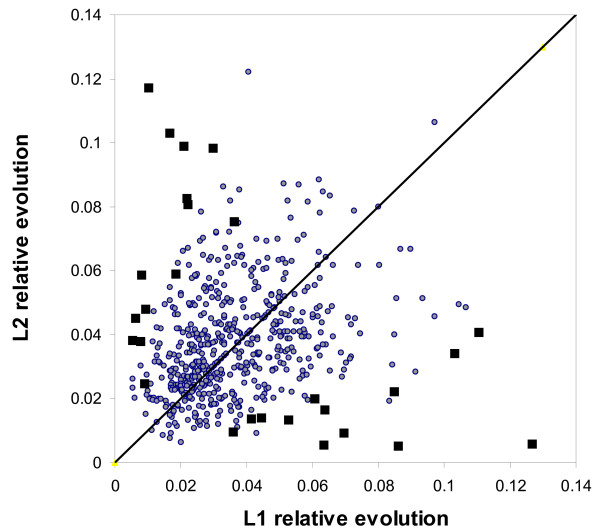
**Symmetric evolution of paralogs**. Scatter plot of relative evolution between *X. tropicalis *peptides and their co-orthologous sequences in *X. laevis*. A total of 578 gene triples with 16 or more highly-conserved positions are shown (see text for details). L1 and L2 refer to co-orthologous genes 1 and 2 in *X. laevis*. The diagonal line represents a null model assuming symmetric evolution of L1 and L2. Black boxes are L1–L2 pairs inconsistent with this model at P < 0.01.

In some scenarios of duplicate gene evolution, one paralog experiences relaxed constraint and/or positive selection for a novel function, while the other evolves under negative selection. To test for such asymmetric evolution, we identified amino acid positions in each LLT triplet that were identical between the *X. tropicalis *peptide and one of its *X. laevis *co-orthologs (and therefore parsimoniously presumed ancestral), but changed in the other *X. laevis *sequence. Figure 2 compares the number of such changes per aligned position for each of the 578 X. *laevis *doublets with 16 or more total changes. In general, changes are evenly distributed between L1 and L2, with 28 pairs (4.8%) showing significant asymmetry at the 1% level relative to a simple neutral model. For this sample size, we would have expected only around six such outliers. Hence, while a few genes do show detectable asymmetric evolution, Figure 2 is generally consistent with a hypothesis of symmetric change under purifying selection [2,25]. This is in agreement with earlier results published by Chain and Evans [28] who detected asymmetric evolution in18 of 290 *X. laevis *paralog pairs (~6%) based on somewhat different statistical criteria.

Gene duplicates have been proposed to exhibit accelerated [31] or slowed [32] evolution, but the effects are subtle and hard to distinguish at the individual gene level. To investigate this effect in bulk we compared amino acid and nucleotide changes between LT orthologs and LL paralogs over an alignment of half a million amino acids. P-distances, 4 DTv (corrected for multiple substitution), and the dN/dS ratio (non-synonymous to synonymous substitutions using all codons) are shown in Table 1. Assuming that synonymous substitution rates are comparable across the genus *Xenopus*, the nucleotide variation provides a simple molecular clock. While LL pairs experienced only ~73% of the nucleotide change of the LT pairs (as the tetraploidization occurred more recently than the *X. laevis*-*X. tropicalis *divergence), they accumulated ~94% as many amino acid changes. Thus, paralogous LL pairs exhibit a relative acceleration of 25–30% more amino acid substitutions (per unit nucleotide change) than orthologous LT pairs. A similar acceleration is detected with the traditional dN/dS ratio (Table 1).

**Table 1:** *X. laevis *paralogs show an enhanced rate of amino acid change relative to *X. laevis*-*X. tropicalis *orthologs.

	**Amino acid substitutions per site**	**Nucleotide transversions per synonymous site**	**Rate of amino acid substitution per unit nucleotide change**	
**Pair**	**P-dist (σ)**	**Corr 4 DTv (σ)**	**P-dist/4 DTv**	**dN/dS (σ)**

**L–T**	0.0598(2)	0.0973(5)	0.615(4)	0.118(1)
**L1–L2**	0.0563(3)	0.0707(6)	0.796(8)	0.147(1)
**L1–L2/L–T**	94.1%	72.7%	129.4%	124.6%

This is demonstrated using both the P-dist/4 DTv measure described in the text, and the conventional ratio of non-synonymous to synonymous substitution rates dN/dS. Both show a 25–30% enhancement in amino acid change. These results are derived from a 513 188 amino acid concatenated, gap-free, multiple sequence alignment (~250 aligned amino acid positions per gene) produced from the 2 135 triplets possessing at least a 50 amino acid aligned block.

**Figure 3:**
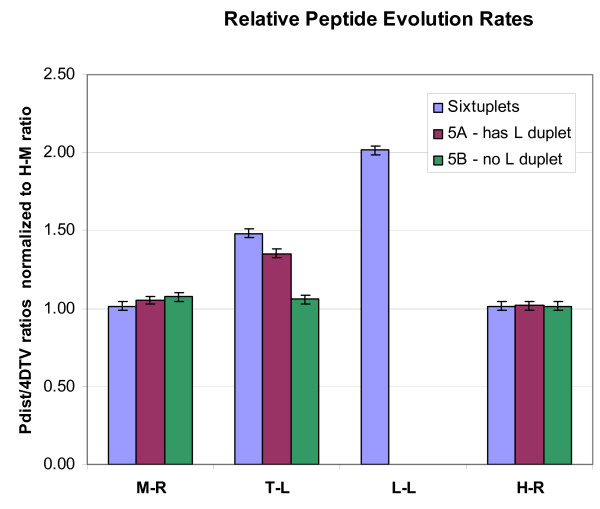
**Normalized peptide to nucleotide evolutionary rates show an accelerated divergence of duplicated *X. laevis *peptides**. The chart shows the ratio of peptide evolution (P-distance) to synonymous transversion rates (4 DTv), normalized by the human-mouse P-distance/4 DTV value of 0.242 ± 0.004, for three sets of multiple alignments corresponding to genes found in single copy in each of human, mouse, rat, and *X. tropicalis*, and two copies in *X. laevis *(sextuplets); pentuplets obtained by randomly selecting one *X. laevis *paralog from each sextuplet (5 A), and pentuplets in which only a single *X. laevis *sequence is known (5B).

Human-mouse-rat (HMR) and LLT nucleotide divergences are roughly comparable (Figure 1), suggesting that the mammalian sequences can provide a control for variations in the evolutionary pattern of change across different gene families. To compare evolutionary patterns in mammals to those in frogs, we identified 904 orthologous LLTHMR sextuplets from these five species totaling 174121 aligned amino acids in conserved blocks (~200 positions per gene). Figure 3 shows the amino acid substitution per synonymous transversion for different subsets of genes, normalized to the human-mouse value. *X. laevis *paralogs show roughly double the rate of peptide change compared with both the human-mouse and mouse-rat matched controls. The intermediate level of LT divergence within retained duplicates is consistent with this effect being confined to the *X. laevis *genes rather than a general feature of frogs that would also accelerate *X. tropicalis *genes. This effect is subtle and requires our large dataset to detect, as it amounts to a little more than one additional amino acid substitution per peptide in *X. laevis *paralogs.

To test whether the acceleration found in *X. laevis *is a feature of retained gene duplicates or simply a feature of all genes in that lineage, we compared genes possessing observed paralogs with apparent single copy genes by identifying two mutually exclusive sets of orthologs from the five species. Set 5A consists of the original sextuplets with one of the *X. laevis *paralogs randomly removed from each gene. The 5B quintuplets each have only a single *laevis *gene with no known recent (4 DTv < 0.2) paralogs. Significantly accelerated evolution in *X. laevis *peptides is found only in genes with a confirmed paralog (Figure 3). For the *X. laevis *genes without recent observed paralogs, the normalized peptide vs nucleotide ratio is 1.11 ± 0.027, much closer to the ratio of 1 seen between the other species. Due to the incompleteness of the EST-derived *X. laevis *gene set we expect some of the 5B genes to have unobserved paralogs in the available *X. laevis *expressed gene set. The observed ratio can be explained if ~20% of the 5B genes have as yet undetected paralogs with the same pattern of evolution as those in 5A.

To study the peptide evolution in *X. laevis *paralogs further, we identified 148401 highly-constrained positions in the six-way LLTHMR multiple alignments, defined as positions with an identical amino acid in human, mouse, rat and at least two of the three frog orthologs. The vast majority of these sites (97.1%) were identical across all six peptides, but 4272 sites (around five residues per peptide) varied in just a single frog sequence. Of these, 26% (1090/4272) occurred in *X. tropicalis*, with ~37% in each of the *X. laevis *paralogs. Thus, even at highly-conserved positions duplicate *X. laevis *genes appear to be accepting additional substitutions eliminated by purifying selection in other species. Similar observations have been made in a number of previous studies (see for example Koonin [2] and references herein).

Are the extra copies of duplicated genes lost in a random fashion following tetraploidization or do different types of genes show different propensities for rentention or loss due, perhaps, to selective constraints? To address this question, we assigned *PANTHER *classification terms to the set of annotated *X. tropicalis *genes based on HHM models [33]. We then grouped these genes into high-level categories of molecular function, biological function, and pathways and compared the relative frequencies of genes within these categories in genes with two retained copies (i.e., member of an LLT triplet) to that of a reference set, using tools and methods developed by Thomas *et al *[34] and described in detail herein. As our reference set we chose all *X. tropicalis *genes with orthologs in *X. laevis*, whether or not a second *X. laevis *co-ortholog is present. No significant difference in frequencies of genes in any of the molecular function categories were found between the two sets (Table 2). This is also true for the biological function and pathway classifications (data not shown) and is in agreement with a similar comparison performed by Morin et al [29].

**Table 2:** Gene content in *X. tropicalis *genes within LLT-triplets compared to the reference set of all *X. tropicalis *genes with *X. laevis *orthologs.

**Molecular function**	**Number within referenceSet**	**Number within LLT triplets**	**Expected based on reference**	**Over- or Under-represented (±)**	**p-Value**
**Transfer/carrier**	123	46	29.11	+	0.06
**Membrane traffic**	165	57	39.05	+	0.107
**Defense/immunity**	90	10	21.3	-	0.146
**Ribosomal**	105	42	24.85	+	0.154
**Hydrogen transporter**	24	14	5.68	+	0.44
**Select regulatory molecule**	479	135	113.36	+	0.625
**Receptor**	309	57	73.13	-	0.787
**Nucleodityltransferase**	35	8	8.28	-	~1
**Double-stranded DNA binding**	10	2	2.37	-	~1
**Transferase**	431	91	102.00	-	~1

Genes were categorized by assigning *PANTHER *classification terms to our *X. tropicalis *gene set by HMM scanning of the peptides and then grouping the terms into high-level categories of molecular function. We were able to assign categories to 6393 genes in the reference set and to 1513 genes in the subset. Out null hypothesis is that the second copy of genes following the *X. laevis *tetraploidization are lost in a random fashion. None of the 10 categories shown here show significant deviation from the null hypothesis, and the remaining molecular categories all have p-values close to 1. All p-values were Bonferroni-corrected for multiple tests.

In addition to sequence evolution, the spatiotemporal expression of duplicated genes could become altered rapidly, generating strong selective pressure to retain both duplicates. This issue has not been addressed in previous studies [28,29]. To begin to investigate expression differences between LL paralogs, we analyzed EST data. While most *X. laevis *genes in our set do not have sufficient counts in any one EST library for a statistically significant determination of differential expression, two large EST sets are available that allow us to address this question: the Osada anterior neuroectoderm library [35] (ANE: 69917 total ESTs; 130 LLT triplets with more than 16 counts) and the NIBB early gastrulation library (EGA: 40476 total ESTs; 40 LLT triplets with more than 16 counts). Under a simple null model for equal expression rates, 53 of the 130 pairs in ANE (40%) have p-values less than 0.01, with only ~1.3 expected under the null hypothesis. At a p-value less than 0.05, we expect 6.5 false positives but observe 68 significant deviations from equal expression. Thus, 40–50% of *X. laevis *genes with sufficient EST data show differential expression in the anterior neuroectoderm, and similarly in the early gastrulation dataset (Table 3). This suggests that many *X. laevis *paralogs have accumulated differential regulatory changes such that they are no longer functionally redundant [6,13,14,32] in terms of their organismal/developmental role. Note that our fraction of doublets showing differential expression is considerably higher than the ~14% found by Morin et al [29].

**Table 3:** Differential expression levels measured using the four largest *X. laevis *EST sets show that a significant fraction doublets show differential expression.

EST library	**ESTs**	**ESTs hitting probes**	**Number of probes hit**	N > = 16	**P < = 0.01**
ANE	69917	9988	1092	130	53
NIBBegast	40476	5424	1199	40	20
NICH_brain1	11005	1278	478	12	2
XGC_Kid1	9662	1504	573	9	3

A total of 2070 matched pairs of antisense "probes" were computed as described in Methods, and applied to the EST data by *in silico *hybridization. Genes with 16 or more hits to ESTs were used to test the null hypothesis that expression levels are the same between paralogs. The four libraries are: ANE (anterior neuroectoderm) [36]; NIBBegast (early gastrulation; Kityama, A, Terasaka, C, Mochii, M, Ueno, N, Shin-i, T, unpublished results); NICH_brain1 (brain; NIH Mammalian collection, unpublished results); and XGC_kid1 (kidney; Heil, O, Neubert, P, Peters, M, Radelof, U, Schneider, D, Schroth, A, Korn, B, Landgrebe, J, unpublished results).

**Figure 4:**
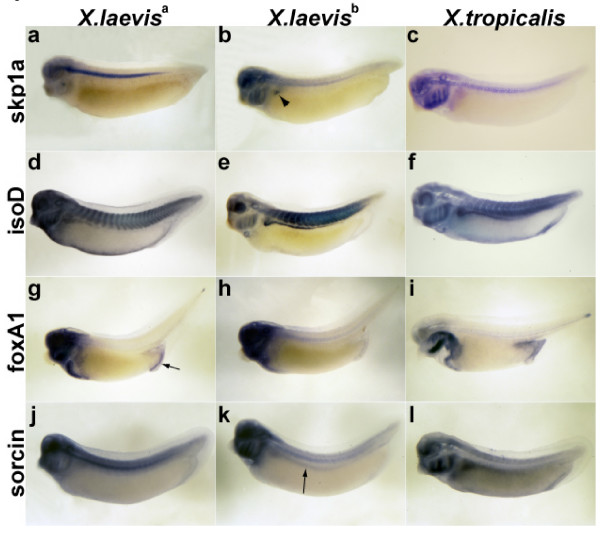
**Expression of specific *X. laevis *paralogs and their *X. tropicalis *ortholog**. Panels depict the expression of skp1a (a-c), isocitrate dehydrogenase (isoD) (d-f), foxA1 (g-i), sorcin (j-l). *X. laevis *paralogs were arbitrarily assigned as a (a,d,g,j) or b (b,e,h,k) and are compared to the *X. tropicalis *ortholog (c, f, i, l). All views are lateral with anterior to the left. Embryos (a-f, j-l) are at stages 31 while embryos (g-i) are at stage 37–38. The arrowhead in (b) indicates kidney expression of skp1a in *X. laevis *paralog b that is not seen in the a paralog. Insets in (d) and (e) magnify somite expression revealing the differential expression between *X. laevis *paralogs ((d) with narrow expression, (e) with broad expression). The arrow in (g) highlights posterior expression of foxA1 seen in paralog a but absent in paralog b. The arrow in (k) indicates weak lateral expression of sorcin in *X. laevis *paralog b that is not seen in paralog a. *X. tropicalis *embryos are shown at a higher magnification than *X. laevis *embryos, reflecting their smaller size.

Higher spatiotemporal resolution of gene expression can be obtained with *in situ *hybridization. Using antisense probes to the highly variable 3' end of transcripts, we examined the expression patterns of four gene triplets in both frog species: the cyclin-associated protein *skp1a*, forkhead box transcription factor *foxA1*, the metabolic enzyme *isocitrate dehydrogenase *(idh), and the calcium binding protein *sorcin*. The spatial expression patterns of the paralogs in *X. laevis *differ from one another and from the pattern of their unique ortholog in *X. tropicalis *(Figure 4).

A striking example is *skp1a*, whose amino acid sequence is 100% identical in all three frog peptides. This peptide is therefore under strong selection across its entire length. One paralog is expressed in the kidney and multiple head structures where the other paralog is either not expressed or only weakly so. These data support studies of other gene pairs in *X. laevis *(and zebrafish) that show subdivided expression patterns relative to single copy counterparts in mammals [13].

## Conclusion

The duplication of an entire genome is a spectacular natural experiment in which tens of thousands of genes are effectively duplicated synchronously, so that each gene has a matched "paralogous" partner with a highly similar or identical sequence and chromosomal context. Subsequent divergence, loss, and rearrangement then gradually erode the signs of duplication. Whole genome duplication can be a powerful evolutionary force, but the polyploidies and subsequent rediploidization that occurred early in the vertebrate and teleost lineages are so ancient (~500 Mya and ~350 Mya, respectively) that the immediate evolutionary response is obscured in modern genomes. Genome tetraploidization occurred more recently in the evolution of *X. laevis *and with extensive genomic and cDNA sequencing available this provides a unique opportunity to analyze a genome in the process of reacting to a recent tetraploidization.

We identify more than 2200 cases in which a single gene in *X. tropicalis *possesses precisely two co-orthologous genes in *X. laevis*, both of which have survived until the present – the largest such collection of orthologs from an animal whole genome duplication. Analysis of such triplets reveals an accelerated evolution, or relaxation of constraint, in the peptides of the *X. laevis *duplicates compared to their orthologs in *X. tropicalis *and other vertebrates. In contrast, *X. laevis *genes for which only one duplicate is retained do not appear to show such acceleration. This is a subtle effect for any single gene, affecting on average only ~1–2 amino acids per peptide, and can only be confidently established by means of the large number of genes available for analysis. The relaxed constraint experienced by retained duplicates is consistent with overlapping/redundant biochemical functions.

The response to genome duplication, however, is more complex than simply relaxing sequence constraints. In one notable example, duplicate *X. laevis *genes produce identical peptides that are also identical to their (single) *X. tropicalis *ortholog. In this case, and in other examples studied with *in situ *hybridization, the *X. laevis *duplicates were found to be expressed in different patterns during development. We looked for other examples of differential gene expression by considering EST counts in deeply sequenced cDNA libraries, and found that a significant fraction (about one third to one half-) of duplicate genes show divergent expression levels in specific tissues. These results are consistent with the subfunctionalization model for the retention of duplicated genes [14,15], in which paralogs acquire complementary coding and/or *cis*-regulatory mutations that leave both copies subject to purifying selection. These changes must occur rapidly, as the lifetime of truly redundant duplicates would be short (few million years) due to (a) the ease with which single nucleotide mutations across a gene can generate a null allele, and (b) the expected nearly neutral selection on such a null allele in the presence of a second locus of identical function.

While whole genome duplications are found in the ancestry of vertebrates, teleost fishes, yeasts, and multiple angiosperm lineages, there are relatively few cases in which a duplicated genome has a natural unduplicated sister sequence that can provide a recent comparative reference. For example, tetrapods can serve as a sister taxon for the study of the teleost duplication, but with a divergence of ~450 million years; for *Arabidopsis*, the related taxa all share either more ancient duplications or their own unique duplications that complicate analysis.

The *X. tropicalis/X. laevis *system provides an ideal testing ground for ideas about whole genome duplication, as the timing of the *X. laevis *tetraploidization is neither "too recent" compared with the lifetime of a duplicated locus, nor "too ancient" for measures of nucleotide variation to have reached saturation. The *X. tropicalis *genome is available in draft form (Richardson et al, unpublished results). As we have shown, the divergence of the two *X. laevis *sub-genomes is extensive, comparable to the divergence between mouse and rat. This suggests that whole genome shotgun approaches would successfully capture the genic regions of the *X. laevis *genome and provide a unique comparative reference for the study of genome evolution.

## Methods

### Identification of *X. laevis *ORFs from DFCI (TIGR) gene indices

We downloaded 39724 tentative clusters (TCs) from the *X. laevis *TIGR gene index version 9.0 (now known as the DFCI indices [36]). All open reading frames (ORFs) in the 5' to 3' direction at least 150 nucleotides long were extracted, translated, and compared against the annotated set of *X. tropicalis *genes (JGI, version 4.1 Department of Energy Joint Genome Institute, 2800 Mitchell Drive, Walnut Creek, CA 94598, USA) using BLASTP [37] with default settings and including hits of E-value 1e-10 or better.

In more than 95% of the cases where an *X. laevis *TC had sequence similarity to an *X. tropicalis *gene, the longest ORF was also the ORF that showed the best BLAST score. In these cases, the longest ORF was selected. In 20% of the remaining 5% the longest ORF still showed similarity, in which case it was selected. Hence, the longest ORF is picked in about 96% of all cases in which the TC has sequence similarity to *X. tropicalis*. In cases where no ORF with sequence similarity exists, the longest ORF was picked, provided that it is at least 300 bases long. Such ORFs are not used in the present analysis. Otherwise, no ORF is annotated for the TC.

In the relatively few remaining cases, we adopted the following heuristics.

In about half the cases in which the longest ORF does not show sequence similarity but a shorter ORF does, the shorter ORF starts immediately at the 5' end, suggesting that the TC is incomplete in the 5' end. In such cases, the incomplete ORF was selected. If the ORF with similarity did not start at the 5' end, we chose the longest ORF if this was longer than 300 bases and the shorter ORF was not. We used this rationale because transposons and low-complexity regions within UTRs occasionally trigger a short ORF with similarity. If the TC has a relatively long ORF, we would suspect that to be the 'real' gene.

In the few remaining cases where both the longest ORF and the homologous ORF are shorter than 300 bases (but longer than 150 bases), we selected the homologous ORF, suspecting that a frame shift or sequencing error could have truncated this ORF.

Many TCs are incomplete at the 5' end. Hence, if the longest ORF started right at the 5' end, we included the entire CDS, even if the translated ORF did not start with a methionine. If the ORF was internal to the TC (i.e., three nucleotides immediately 5' of the ORF start translate into a stop codon), we interpret the gene as complete with 5' UTR. We report only on the CDS from the first ATG if it is longer than 150 nucleotides, unless the translated ORF has clear hits to a *X. laevis *gene at least 20 amino acids upstream of the first methionine, in which case the entire frame will be reported. The latter scenario could conceivably result from a sequencing error.

This annotation procedure resulted in 24674 candidate transcripts and peptides, 20825 of which show significant (< 1e-10) similarity to human genes. A total of 11711 (47.4%) were deemed partial by the above criteria. Some of the transcripts might be alternatively spliced versions of the same gene, which we identified by having evolutionary distances of 0, or close to 0. To reduce the number of shorter forms of alternatively spliced genes we applied the following filtering procedure. From the all-against-all Smith-Waterman alignment of the peptides described below, we evaluated 4DS distances, i.e., the fraction of four-fold degenerate third codon positions showing a nucleotide substitution. For all pairwise alignments with at least 25 conserved four-fold degenerate codon positions and not a single substitution observed, the shorter of the transcripts was marked as a short alternative splice form, and excluded from further analysis. A total of 1777 transcripts were filtered out in this manner, leaving 22897 *X. laevis *genes, 19211 of which showed similarity to human genes, 19598 to *X. tropicalis *genes, and 20223 to either *X. tropicalis *or human. These 20223 peptides and corresponding CDS sequences were used in subsequent analysis.

### Identification of LLT orthologous triples

We aim to identify unambiguous sets of L1–L2-T triplets where L1 and L2 are the only two known recent copies in *X*. *laevis *and have an evolutionary distance consistent with originating from the whole genome duplication epoch, whereas the *X. tropicalis *version T does not have any known recent paralogs. We first performed all-against-all double affine Smith-Waterman alignments of the peptides in *X. laevis *and *X. tropicalis *using a TimeLogic DeCypher system (Active Motif, Inc., 1914 Palomar Oaks Way, Suite 150, Carlsbad, CA. 92008) with BLOSUM62 scoring matrix, gap opening penalty -15, gap extension penalty -2 until gap size 10, with no additional extension penalties. We identified the conserved four-fold degenerate amino acids within the alignments, extracted the corresponding codons in the underlying DNA sequence and calculated the 4 DTv distances (*D*_4*DTv*_) between each aligning pair as the fraction of four-fold degenerate (4D) third codon positions in which transversions are observed to have occurred. This provides a measure of the evolutionary distances between genes that is largely independent of the gene families, unlike measures based on peptides. *D*_4*DTv *_ranges from 0 for recently duplicated peptides, to ~0.5 for paralogs that are so old that third codon nucleotides have essentially been randomized. Assuming that transversions occur independently, with equal probability at all, 4D sites, we can correct for multiple substitutions using the simple formula:

*D*_4*DTv*,*corr *_= -1/2ln(1-2*D*_4*DTv*_)

In addition, we calculated the fraction of 4D sites that had experienced any substitution, transition or transversion, *D*_4*D*_. This distance measure gives better resolution for recent paralogs.

**Figure 5:**
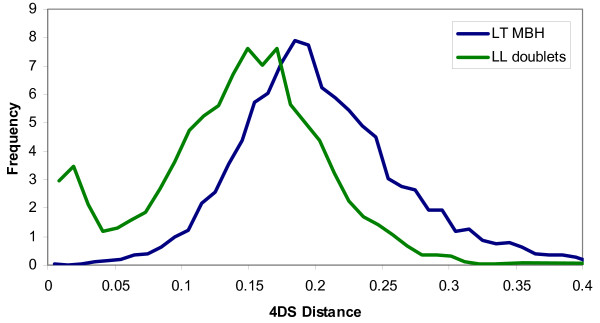
**4DS distances identify genome duplication event**. Histograms of the 4DS distances for the 9905 mutual highest scoring L-T pairs (blue line) as well as for the 3358 unambigous L-L pairs (red bars). The L-L pairs with 0.05 < 4DS < 0.25, peaking around 0.16, are selected as originating from the genome duplication event.

Next, we performed a single-linkage clustering of all *X. laevis *genes hitting other *X. laevis *genes with 0 ≤ *D*_4*DTv *_≤ 0.2. We disregarded alignments with fewer than 25 conserved 4D sites as we cannot determine reliable 4 DTV distances for such proteins, and these are either too incomplete or evolving too fast at the peptide level for the purpose of our analysis. A total of 3358 of the resulting clusters had exactly two members. The distribution of 4 DTv distances in these pairs is shown in Figure 1a. Indeed, the peak at around 4 DTV ~0.067 indicates that the majority of these paralogous pairs were created at a single epoch, that of the *X. laevis *whole genome duplication. However, some of the pairs with 4 DTV values close to 0 are likely to represent cases of more recently duplicated paralogs, which cluster as a 2-member cluster because the paralog from the duplication epoch has either been lost or is not represented in the EST set. The median and mean number of conserved 4D sites for the candidate doublets are 92 and 112, respectively, so the typical resolution will be of the order ~0.01 in 4 DTV and 4DS. Due to the short evolutionary distance, "discreteness effects" are visible in the 4 DTV distribution, where, for the gene pairs with only ~25 conserved 4D sites a difference between 0 and 1 observed substitution translates into a 4 DTV distance of 0 vs 0.04, a considerable fraction of the duplication epoch. For better resolution we use the *D*_4*DS *_to select the candidate gene pairs from the duplication event. Figure 5 shows the distribution functions of these distances for the L-T orthologs and L-L doublet candidates. The L-L distribution appears bimodal, with peaks around 4DS = 0 (recent duplicates) and 4DSS ~0.16 (from the epoch of the whole genome duplication). From this insight, we conservatively selected all pairs with 0.05 < = 4DS < = 0.25 as our set of gene pairs from the epoch of genome duplication that have no other known recent paralogs. This amounts to 2875 doublets.

Of the 9905 mutual best hitting *laevis*-*tropicalis *pairs 9574 – almost 97% – have 4 DTV < = 0.2. These genes are almost certainly truly orthologous pairs. Of these, 843 have one or more recent paralog in *X. tropicalis *as defined by having 4 DTV < 0.2 to a homologous *X. tropicalis *gene. We eliminated these genes from consideration, as the functional evolution is more difficult to interpret when multiple paralogs are present. For each of the remaining 8731 pairs, we identified an unambigous LLT triplet if the *X. laevis *gene was a member of one of the 2875 doublets previously identified. This method resulted in 2218 unambiguous LLT triplets used in the study. The CDS and peptide sequences of these triplets, along with identifiers mapping the *X. laevis *genes to their corresponding TCs are available in Additional file 1. The sequence similarity between a pair of *X. laevis *CDS sequences in a triplet is typically about ~93%, whereas in the less conserved corresponding UTR regions it is no more than 85–87%, with several gaps in the alignments. Clearly, paralogs from the duplication events are sufficiently distinguishable for correct assembly of the EST clusters. In addition, the distinct UTR regions allows for selection of unique probes for our *in situ *hybridizations, as described later.

### Estimate of the fraction of retained duplicate genes

We made two rough boundary estimates of the fraction of originally duplicated genes that has been retained in the modern *X. laevis*. First, we have seen in the previous section that of 8731 L-T orthologs, 2218 were found to have a second L co-ortholog, which would suggest a retention fraction of *f *= 2218/8731 = 0.25. However, this must be a minimum estimate as some co-orthologs will inevitably be missed due to the incompleteness of the *X. laevis *gene set. At the other extreme we can assume that for any L-T orthologous pair, the probability *p*_*miss *_of missing an existing co-ortholog due to incompleteness is 1-N_EST,L_/N_tot,L_, where N_EST,L _is the number of *X. laevis *genes in our EST-based set and N_tot,L _is the total (unknown) number of genes in the *X. laevis *genome, which can be expressed in terms of the size N_tot,T _of the *X. tropicalis *genome, if we assume that these two genomes differ mainly due to the presence of duplicate genes. In that case we have N_tot,L _= (1+*f*) N_tot,T_, where *f *is the retention fraction. Combining this with the expression for *p*_*miss *_above, and using the approximation N_EST,L _= N_tot,T _= 20000 genes, we get *p*_*miss *_= *f*/(1+*f*). The total number of L-T orthologs with retained co-orthologs, corrected for incompleteness is then 8731 *f *= 2218+(8731-2218)*p*_*miss*_. Substituting *p*_*miss *_and solving for *f *we get *f *= 0.5, that is, half the original duplicates are still present. This is likely to be an upper estimate, as the calculation of *p*_*miss *_assumes that any gene has an equal possibility of being in the *X. laevis *EST set, whereas in reality, once we have observed the presence of one co-ortholog in this set, the other co-ortholog, if it exists, could well have a larger-than-average probability of being included as well as this set are biased towards highly expressed genes.

Based on these estimates, we conclude that at least 25% and at most 50% of the duplicated genes in *X. laevis *have been retained. Interestingly, from the study of the quintuplets in the results section, we argued that we could account for the observed patterns of acceleration if 20% of the 5B (single-copy) genes had undetected co-orthologs. This would be consistent with a retention rate of *f *~ 40%.

### Multiple sequence alignment and peptide evolution analysis

We performed multiple sequence alignments of the LLT triplets using the clustalW program [38] with default settings, and extracted blocks of gap-free aligning sequence flanked by fully conserved amino acids and allowing no more than four consecutive positions of non-conserved amino acids within each block. A total of 2 135 of the triplets had a least 50 amino acids in such highly-conserved blocks, which concatenated into 513 188 amino acid residues for which combined P-distances (i.e., fractions of differing amino acids) and 4 DTV distances could be evaluated. The results are shown in Table 1.

### Symmetric evolution

For aligned LLT triplets, we extracted highly-conserved gap-free blocks. In these regions, we examined all positions where either L2 and T had an identical amino acid residue but L1 did not, or L1 and T were identical and L2 was not. Let (N1, N2) denote the total counts of such positions for each triplet. These are candidate positions for assymetric evolutionary changes. The relative evolution parameters shown in Figure 2 are N1/N, N2/N, where N is the total number of aligned amino acids. Our null hypothesis of no assymmetric evolution assumes that N1 and N2 are drawn from a binomial distribution with mean (N1+N2)/2 and probability of 0.5. For each observed number (N1, N2), we then calculated the p-value as the probability of observing a result at least as skewed, under the null hypothesis, i.e.:

P=2∑N=0N1PBin(N,N1+N2)
 MathType@MTEF@5@5@+=feaafiart1ev1aaatCvAUfKttLearuWrP9MDH5MBPbIqV92AaeXatLxBI9gBaebbnrfifHhDYfgasaacH8akY=wiFfYdH8Gipec8Eeeu0xXdbba9frFj0=OqFfea0dXdd9vqai=hGuQ8kuc9pgc9s8qqaq=dirpe0xb9q8qiLsFr0=vr0=vr0dc8meaabaqaciaacaGaaeqabaqabeGadaaakeaacqWGqbaucqGH9aqpcqaIYaGmdaaeWbqaaiabdcfaqnaaBaaaleaacqWGcbGqcqWGPbqAcqWGUbGBaeqaaOGaeiikaGIaeeOta4KaeeilaWIaeeOta4KaeeymaeJaey4kaSIaeeOta4KaeeOmaiJaeiykaKcaleaacqWGobGtcqGH9aqpcqaIWaamaeaacqWGobGtcqaIXaqma0GaeyyeIuoaaaa@4519@

Where *P*_*Bin*_(N, N1+N2) is the binomial probability function. This method can only detect significantly skewed (i.e., ~10 or more AA changes) evolution of peptides. That is, we do not have the statistical power to identify cases where a single change at a strategic site changes the function of the peptide.

### Differential expression

To evaluate the relative expression of members in *X. laevis *doublets we aligned the nucleotide sequence in the 2135 confirmed doublets using BLASTn [37] with a cutoff in e-value of 1e-100. If the aligning sequence, stripped for gaps, was longer than 199 bases, we picked this sequence pair as a probe-set against which ESTs from any library can be aligned. By this method we were able to construct 2070 pairs of probes. The members of each pair are sufficiently distinct from each other (mean and median ~92.7% identity) that it can be unambiguously identified which of the two probes is the correct match for a given EST. As quite a few ESTs contain undetermined bases, and SNPs could be present, we don't always see a 100% match. We define all hits to one of the members probe-set better than 98.5% as a match.

To test whether *X. laevis *pairs differed significantly in expression level, we performed a statistical analysis similar to that performed to detect asymmetric evolution in peptides. For each pair of EST hits (N1, N2) where N1 and N2 are the number of ESTs compatible with probe 1 and 2, respectively, we calculated the probability of the observed results or worse under the hypothesis that each gene in the probe pair are equally expressed, i.e., had an equal probability of being assigned an EST. This probability, evaluated using the normal approximation to the binomial distribution, constitutes a p-value for each of these 130 probe pairs.

### Identification of 301 candidate doublets from zebrafish whole genome duplication

The zebrafish doublets shown in Figure 1a were determined as follows: the Ensembl [39] models v. 24.4.1 were aligned to each other and to the Ensembl models v. 26.35.1 for human on Timelogic Decypher™ using the same parameter settings as for the frog aligments, and 4 DTv distances were determined for each pair with 25 or more 4D codon sites. A single-linkage clustering of paralogs hitting each other with a P score < 10^-20 ^was then performed, and all clusters with more than eight members were rejected as promiscuous genes. On the remaining set, we performed a mutual-best hitting algorithm excluding hits with (a) 4 DTv distance < 0.25 (recent paralogs), and (b) genes on the same chromosome within 5 megabases from each other. These hits are from tandem duplications or recent paralogs and hence not candidates for the zebrafish whole-genome duplication. From the remaining pairs, we removed pairs in which (a) both members had different orthologs in human, as determined by mutual best hits (paralogs preceding the human-fish lineage split), and (b) pairs with no human orthologs (and hence undatable). In the remaining cases, we performed multiple sequence alignments of the human-ZF1-Zf2 triplets and calculated the P-distances in conserved, gap-free blocks. We then retained the pairs in which the Zf-Zf2 P-distance was shorter than either human-Zf1 or human-Zf2, as these are likely to be a result of a duplication event that happened after the human-Zf split. The 4 DTv distance distribution for the 301 remaing pairs is shown in Figure 1a.

### Comparison to other vertebrates

We compared the sequence evolution rates of the LLT triplets to human, mouse, and rat genes in the following manner. For each of these three species, we downloaded the set of Ensembl gene models and, using only the longest gene at each locus, we identified blocks of conserved synteny between each pair of species using a PERL implementation of the following algorithm: for the first pairwise aligment of genes in the proteomes of the two species, the gene locations on the chromosomes is recorded and a one-pair segment of conserved synteny is defined. Subsequent gene pairs either defines new segments, or, if the genes in both species are located within a specified maximum distance from a gene pair in an existing segment, the pair is added to that segment. If a pair can be added to two segments, these segments are joined into a larger segment of conserved synteny. After traversing all alignments, we have a set of conserved syntenic regions, on which we can impose a minimum member limit (typically three pairs) to removed spurious regions. In the vertebrates, regions of conserved synteny can extend over several hundred genes. A gene in one species can, and usually does, form part of more than one block of conserved segments. However, the longest such block usually defines the orthologous region, whereas smaller blocks are remnants of either ancient genome duplications or recent segmental duplications. For the purpose of this study, we retained only the strictest set of orthologs, confirmed by the longest block of conserved synteny covering the area, and excluding all genes found to be members of a tandem duplicated family, in order to avoid mis-identified orthologs. For human-mouse, ~95% of the synteny-confirmed orthologous pairs are also mutual best hits to each other. A total of 9852 tropicalis genes have synteny-confirmed orthologs with at least one human, mouse, or rat gene, and 5475 have synteny-confirmed orthologs in all three. The 4 DTV distributions for orthologous pairs defined in this manner are shown in Figure 1b. It is seen that they indeed peak around characteristic values that reflects the evolutionary distance between the species. By this measure, *laevis*-*tropicalis *and the two *X. laevis *doublets are at an intermediate evolutionary distance between that of mouse-rat and mouse-human.

In 1039 of the LLT triplets, the *X. tropicalis *gene had synteny-confirmed orthologs to human, mouse, and rat and were used to construct clusters of six genes containing two *laevis *co-orthologs and their corresponding single *tropicalis*, human, mouse, and rat orthologs.

After multiple sequence alignment, 904 of the sextuplets showed conserved blocks of at least 50 amino acids among all six peptides in the same manner defined above for the triplets.

### Test for EST artifact in peptide evolution

To rule out the possibility that the higher rate of peptide evolution in *X. laevis *is simply an artifact caused by EST sequencing errors, we performed the same analysis on the subset of 339 sextuplets for which the *X. laevis *doublets were both based on TCs assembled from 12 or more ESTs. For such clusters, sequencing errors associated with individual ESTs will generally be corrected by overlapping ESTs used in the consensus sequence. The peptide evolution to 4 DTV ratio stayed the same in this subset, however, as well as for an even more restricted subset of 158 doublets with 24 or more ESTs (data not shown).

### *In situ *hybridization

We generated digoxigenin labeled RNA probes and performed whole mount *in situ *hybridization as previously described for *X. laevis *and *X. tropicalis *embryos [40,41]. For *X. tropicalis*, we generated probes using the entire length of the cloned insert. In order to detect paralog specific expression in *X. laevis*, we generated probe only from the 3' UTR, as outlined in Table 4.

**Table 4:** Description of triplets selected for *in situ *hybridizations

Gene	Species paralog	Clone	Cut/transcribe
Skp1a	L1	IMAGE:6946267	*Spe*I/T3
Skp1a	L2	IMAGE:7202221	*Xmn*I/T7
Skp1a	*X. tropicalis*	IMAGE:6995134	*Eco*RI/T7
Isocitrate dehydrogenase	L1	IMAGE:3474748	*Acl*I/T7
Isocitrate dehydrogenase	L2	IMAGE:5542876	*Acl*I/T7
Isocitrate dehydrogenase	*X. tropicalis*	IMAGE:6995129	*Eco*RI/T7
foxA1	L1	IMAGE:5572849	*Stu*I/t7
foxA1	L2	IMAGE:4203644	*Bstx*I/t7
foxA1	*X. tropicalis*	TGas068H09	*Cla*I/T7
Sorcin	L1	IMAGE:4957318	*Sac*I/T7
Sorcin	L2	IMAGE:7204932	*Sac*I/T7
Sorcin	*X. tropicalis*	IMAGE:4461879	*Cla*I/T7

In some instances, paralog probes in *X. laevis *detected no significant expression differences and were set aside for this analysis (data not shown). However, as shown in Figure 4 some probes identified different expression patterns for the two paralogs in *X. laevis *(also indicating that they were a paralog specific probe set). In each case to confirm expression patterns, over three dozen embryos were stained for each probe in three different *in situ *hybridization experiments. Expression patterns shown in Figure 4 are representative and were consistently seen across all of the embryos analyzed.

## Authors' contributions

UH and DR conceived the study and performed the bioinformatics data analysis. DR and UH drafted the paper. MK and TG performed the *in situ *hybridizations. PR led the sequencing of the *X. tropicalis *genome and RH supervised experimental work. All authors read and approved the final manuscript.

## Supplementary Material

Additional file 1 **Nine columns in TAB-separated format**. Each of the 2 218 rows contains the CDS and peptide sequence for the LLT triplets as follows. Columns 1–3 contain a unique identifier, the CDS nucleotide, and the peptide sequence, respectively, for one of the *X. laevis *genes in the triplet. In the same manner, columns 4–6 contain identifier and sequences for the other *X. laevis *gene in the triplet, whereas columns 7–9 contain this information for the *X. tropicalis *member of the triplet. The *laevis *identifiers are those of the DFCI gene indices of the corresponding EST clusters, whereas the *tropicalis *ids are unique identifiers internal to our database. Click here for file
